# Effect of teacher–student relationship on academic engagement: the mediating roles of perceived social support and academic pressure

**DOI:** 10.3389/fpsyg.2024.1331667

**Published:** 2024-06-20

**Authors:** Xiangliang Liu

**Affiliations:** College of Geography and Environment, Shandong Normal University, Jinan, China

**Keywords:** academic engagement, academic pressure, perceived social support, teacher-student relationship, university students

## Abstract

Although previous research has established that a strong teacher–student relationship can enhance students’ academic engagement, the mechanisms underlying this effect remain less explored. Therefore, this study examined the mediating roles of perceived social support and academic pressure in the association between teacher–student relationship and academic engagement. A survey involving 1,058 Chinese university students was conducted, with teacher–student relationship, perceived social support, academic pressure, and academic engagement being the evaluated factors. The results of structural equation modeling revealed that (a) teacher–student relationship directly and positively associated academic engagement, (b) teacher–student relationship indirectly and positively associated academic engagement through perceived social support, and (c) teacher–student relationship indirectly and positively associated academic engagement through both perceived social support and academic pressure. These results indicate that perceived social support and academic pressure are the primary factors mediating the effect of teacher–student relationship on academic engagement among university students.

## Introduction

Academic engagement is a state of mind in which students not only experience a fulfilling and enjoyable learning journey but also have a strong sense of identification with their studies and the ability to maintain focus and energy throughout the learning process (Lam et al., 2012; Doggrell, 2023). Academic engagement is a key predictor of students’ academic performance, which makes academic engagement a crucial metric for evaluating learning quality (Moreira et al., 2013; Sun, 2014; Silva and Almeida, 2023), with China’s increasing emphasis on the quality of higher education and the promotion of positive mental health education, scholars have gradually paid attention to and comprehensively studied academic engagement, particularly because such engagement encapsulates the positive learning state of university students.

According to self-determination theory (Ryan and Deci, 2000), positive teacher–student relationships was helpful to satisfy individuals’ basic needs, such as autonomy, relatedness and competence, which was the foundation for students’ study and resulting in more academic engagement (Li, 2022). Study have suggested students with positive teacher–student relationships could perceived more social support from their teachers and less academic pressure, which help them to spend more time on learning (Van Herpen et al., 2020; Qonita et al., 2021). Based on the self-determination theory, the present study aimed to test the association and its mechanism between teacher–student relationships and academic engagement.

## Effect of teacher–student relationship on academic engagement

The relationship between teachers and students has been identified as a significant predictor of academic engagement in past studies. Feedback and positive evaluations from teachers have been shown to foster a positive academic self-concept in students (Lin et al., 2001), which stimulates their interest and heightens their engagement levels (Chen et al., 2021; Pham et al., 2022). A meta-analysis by Roorda et al. (2011) also indicated that when students maintain a harmonious relationship with their teachers, the students are more inclined to participate actively in class activities. Moreover, other studies have demonstrated the benefits of a positive teacher–student relationship, highlighting its role in enhancing students’ sense of security, fostering a conducive learning atmosphere, and setting a solid foundation for sustained engagement (Hamre and Pianta, 2001; Zee and Roorda, 2018). Although the aforementioned studies have explored the effect of teacher–student relationship on students’ academic engagement, they have focused on primary and secondary school students; studies focusing on university students are limited. Accordingly, the aim of the present study was to extend the current understanding by exploring the effect of teacher–student relationship on university students’ academic engagement.

### Mediating role of perceived social support

Social support is a valuable resource influencing physical and mental health as well as psychological and social adaptation, and this resource ultimately contributes to personal achievement. Essentially, social support represents the benefits that individuals reap from their social connections and helps them to mitigate psychological stress, alleviate mental strain, and enhance social adaptability (Tsuno and Yamazaki, 2012). Social support is typically classified into two categories: (a) perceived social support, which refers to the social resources that individuals believe they can access, and (b) objective social support, which denotes the actual support received through established relationships (Wang et al., 2018; Chang et al., 2020). Perceived social support often depends on objective social support; however, research on higher education has focused more on perceived social support (Lee, 2018). Sources of perceived social support for university students can encompass family, friends, teachers, communities, and affiliated social groups. Notably, support from family and friends considerably associates students’ academic outcomes. A higher degree of perceived social support from these groups is associated with better overall adaptation to university life (Mahzan et al., 2014), possibly because students feel backed in their academic pursuits.

Students with greater access to high-quality social support typically exhibit better health and receive more emotional support from peers, which is instrumental in fostering academic adaptation and engagement (Elffers et al., 2012; Elsaesser et al., 2018). For students attending residential universities, direct parental support might be less accessible; thus, the importance of support from teachers and classmates is amplified for such students. In particular, teachers offer more academic guidance than peers do; therefore, the support of teachers is crucial for improving academic engagement. A stronger teacher–student relationship translates to increased social support, which leads students to perceive enhanced support, thereby elevating their engagement levels. Accordingly, perceived social support appears to mediate the association between teacher–student relationship and academic engagement.

The association between perceived social support and students’ academic engagement remains a topic of debate. Although some studies have reported no substantial effect of perceived social support on engagement (Shi, 2005; Cirik, 2015), others have identified a positive association between high levels of perceived support and enhanced engagement (Lam et al., 2012). Considering these contrasting viewpoints, additional studies are warranted to elucidate the effect of perceived social support on students’ academic engagement.

### Mediating role of academic pressure

Academic pressure can be defined as the psychological discomfort and tension experienced by students during the learning process (Xu et al., 2010; Qonita et al., 2021). Moderate academic pressure can stimulate students’ enthusiasm for learning, thus improving their academic engagement and performance. However, excessive academic pressure can lead to adverse reactions, such as fatigue and an aversion to learning; it can even cause physiological discomfort in students, which can negatively associate their academic engagement and performance (Callicott and Park, 2003; Linnenbrink-Garcia and Pekrun, 2011; Zhang, 2014). Academic pressure is a comprehensive, chronic, and diffuse source of stress and thus permeates all aspects of university students’ academic activities. In the context of Chinese higher education, students often face immense pressure to excel academically because academic excellence is closely tied to postgraduate opportunities and employment prospects (Ni et al., 2013). Many students thus experience heightened academic pressure, which potentially associates their academic engagement negatively. However, having supportive university teachers endowed with relevant advanced professional skills can alleviate these pressures (Roorda et al., 2011; Moreira et al., 2018). Positive teacher–student relationships facilitate more frequent teacher–student interactions and thus enhanced academic guidance, which lead to reduced perceived academic pressure and improved academic engagement.

### Combined mediating role of perceived social support and academic pressure

Several studies have suggested that perceived social support can effectively alleviate the academic pressure faced by university students (e.g., Mahzan et al., 2014; Lee, 2018). According to the student integration model, social support facilitates students’ seamless integration into the academic environment, thereby increasing their satisfaction level and active participation in learning, which can increase their commitment to completing their academic pursuits (Tinto, 1975). The literature indicates that social support is negatively associated with academic stress (Khan et al., 2015) and that social support is a major predictor of academic stress (Malla et al., 2015). Support from family members (Glozah and Pevalin, 2014), friends, teachers, and significant others (Fernández-González et al., 2015) can moderate students’ academic pressure. However, in the university context, students require support in the form of advanced professional knowledge to overcome their learning difficulties, which means that social support from family members and friends is insufficient in this context. Teachers often play a pivotal role in the university context. They are uniquely positioned to offer specialized guidance to help students to overcome specific learning challenges, thereby reducing the stress arising from these challenges. Thus, a positive teacher–student relationship can enhance perceived social support, thereby leading to decreased academic pressure and increased academic engagement.

### Present study

**Figure 1:**
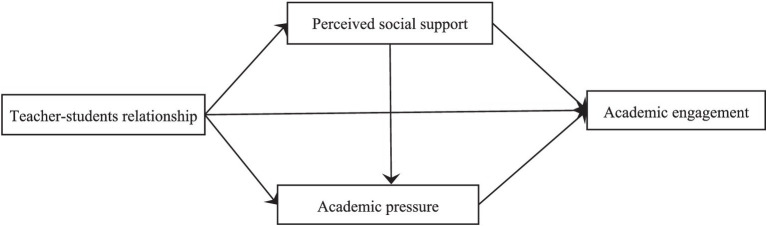
Conceptual model of this study.

Although previous studies have found significant associations among teacher–student relationship, perceived social support, academic pressure, and academic engagement (Tsuno and Yamazaki, 2012; Qonita et al., 2021; Pham et al., 2022), we knew little about intrinsic relationship mechanism among them. To test the intrinsic relationship mechanism among teacher–student relationship, perceived social support, academic pressure, and academic engagement tended to be helpful for the interventions for improving university students’ academic engagement, and therefore, the aim of the present study was to examine the association between teacher–student relationship and academic engagement among university students, with particular attention on the mediating roles of perceived social support and academic pressure. On the basis of the aforementioned literature review, we formulated a conceptual model (Figure 1). By using this model, we propose the following hypotheses:

- Teacher–student relationship could positively associate academic engagement (H1).
- Teacher–student relationship could positively associate academic engagement through perceived social support (H2).
- Teacher–student relationship could positively associate academic engagement through academic pressure (H3).
- Teacher–student relationship could positively associate academic engagement through both perceived social support and academic pressure (H4).

## Methods

### Participants

In this study, 1,100 university students from * * city were recruited to participate in a questionnaire survey. Copies of the questionnaire were sent to these students, and 1,058 valid questionnaires were collected; thus, the valid response rate was 96.18%. Among the participants who provided valid responses [average age = 19.63 years; standard deviation (SD) = 1.13], 596 were men (56.3%) and 462 were women (43.7%). Moreover, 597 participants (56.4%) had an urban registered residence, and 461 participants (43.6%) had a rural registered residence. The participants’ average annual household income was 5.67 (SD = 2.24); the income was assessed on a 10-point scale, with 1 indicating 0 yuan [51 participants (4.8%)], 2 indicating <2,000 yuan [17 participants (1.6%)], 3 indicating 2,000–5,000 yuan [74 participants (7.0%)], 4 indicating 5,000–10,000 yuan [190 participants (18%)], 5 indicating 10,000–30,000 yuan [231 participants (21.8%)], 6 indicating 30,000–50,000 yuan [117 participants (11.1%)], 7 indicating 50,000–100,000 yuan [148 participants (14.0%)], 8 indicating 100,000–150,000 yuan [98 participants (9.3%)], 9 indicating 150,000–200,000 yuan [63 participants (6.0%)], and 10 indicating >200,000 yuan [69 participants (6.5%)].

### Ethics statement

This study was approved by the Human Research Ethics Committee of XYZ University and conducted in accordance with the ethical principles of the Declaration of Helsinki and its later amendments. Written informed consent was obtained from all participants.

### Survey medium

Because of the constraints posed by the COVID-19 pandemic, all questionnaires were administered online. Students had the flexibility to use devices such as computers, iPads, or smartphones for questionnaire completion. The homeroom teachers provided guidance and support throughout the questionnaire-filling process, with the average survey duration being approximately 20 min. Moreover, informed consent was obtained from all parents for their children’s participation in this study.

### Measurements

#### Teacher–student relationship

This study used the Chinese version of the Student–Teacher Relationship Scale (Wang, 2016) — the original version of which was developed by Piana et al. (1994) — to evaluate teacher–student relationship. This scale contains 22 items covering four dimensions, namely avoidance, conflict, intimacy, and attachment. The avoidance dimension comprises four items (e.g., “I do not want to have contact with the teacher”), the conflict dimension comprises nine items (e.g., “Teachers often argue with their students”), the intimacy dimension comprises four items (e.g., “I am very concerned about my teacher”), and the attachment dimension comprises five items (e.g., “The teacher is very fair to the students”). The items of the aforementioned scale are rated on a 5-point Likert scale ranging from 1 (*completely disagree*) to 5 (*completely agree*). A higher total score indicates a more positive teacher–student relationship. In this study, the Cronbach’s *α* value for the Student–Teacher Relationship Scale was 0.92.

#### Perceived social support

We used the revised version of the Perceived Social Support Scale (Jiang, 1999) — the original version of which was developed by Zimet et al. (1988) — to measure perceived social support. This scale contains 12 questions covering three dimensions, namely family support (e.g., “I can receive emotional help and support from my family when needed”), friend support (e.g., “I can rely on my friends when difficulties arise”), and other support [e.g., “some people (leaders, relatives, or colleagues) will appear next to me when I encounter problems”] dimensions. The items of the aforementioned scale are rated on a 7-point Likert scale ranging from 1 (*strongly disagree*) to 7 (*strongly agree*). The sum of the scores of all items represents the respondent’s level of perceived social support, and a higher score indicates a higher level of perceived social support. In this study, the Cronbach’s *α* value for the Perceived Social Support Scale was 0.98. And the scale also had good validity: *χ^2^* = 804.11, *df* = 151, *p* < 0.001, RMSEA = 0.064, TLI = 0.94, and CFI = 0.96.

#### Academic pressure

We used the China College Student Psychological Stress Scale (Liang and Hao., 2005) to measure the participants’ academic pressure. This scale contains 16 items, which are rated on a 2-point Likert scale ranging from 1 (*I have not experienced stress*) to 2 (*I have experienced stress*). The total score indicates the degree of academic pressure experienced by the respondent. A higher score indicates a higher degree of academic pressure. In this study, the Cronbach’s *α* value for the aforementioned scale was 0.61.

#### Academic engagement

This study used the Chinese version of the Academic Engagement Scale (Shi et al., 2008)—the original version of which was developed by Schaufeli et al. (2002) — to measure the participants’ academic engagement. This scale contains 17 items that cover three dimensions, namely vitality, dedication, and focus. The vitality dimension comprises six items (e.g., “When studying, I feel energetic”), the dedication dimension comprises five items (e.g., “When studying, even if I am mentally fatigued, I can recover quickly”), and the focus dimension comprises six items (e.g., “I feel very happy when I devote myself wholeheartedly to learning”). The scale items are rated on a 7-point Likert scale ranging from 1 (*never before*) to 7 (*always*/*every day*). In this study, the Cronbach’s *α* value for the Academic Engagement Scale was 0.98.

### Data processing

SPSS 23.0 was used for descriptive analyses, and Mplus 7.3 was used for structural equation modeling (SEM). The following indices were used to assess the model’s fit: *χ^2^*/*df*, root mean square error of approximation (RMSEA), comparative fit index (CFI), and Tucker–Lewis index (TLI). If the *χ^2^*/*df* value is <3, RMSEA is <0.08, and CFI and TLI are >0.90, then the model under assessment has good fit with the collected data. In addition, gender, registered residence, age, and annual household income were used as control variables in the analyses.

## Results

### Descriptive statistics and correlation analysis

Table 1 presents the means and SDs for teacher–student relationship, perceived social support, academic pressure, and academic engagement; this table also presents the associations between these variables. Teacher–student relationship was positively associated with perceived social support and academic engagement but was negatively associated with academic pressure. Perceived social support was negatively associated with academic pressure but was positively associated with academic engagement. Academic pressure was negatively associated with academic engagement.

**Table 1:** Descriptive statistics and correlations for the variables.

	1	2	3	4	5	6	7	8
Gender	1							
Registered residence	−0.01	1						
Age	−0.10^***^	0.01	1					
Annual household income	0.10^***^	−0.28^***^	−0.04	1				
Teacher-students relationship	0.01	−0.10^***^	−0.06^*^	0.10^***^	1			
Perceived social support	0.06^*^	−0.07^*^	0.01	0.16^***^	0.49^***^	1		
Academic pressure	0.02	−0.06^*^	−0.01	−0.02	−0.08^**^	−0.10^***^	1	
Academic engagement	−0.08^**^	−0.03	0.07	0.03	0.37^***^	0.43^***^	−0.13^***^	1
M	–	–	3.63	–	88.93	67.42	5.79	87.64
SD	–	–	1.13	–	13.39	13.54	2.34	22.86

Gender (0 = male; 1 = female); registered residence (0 = urban registered residence; 1 = rural registered residence). ^*^p < 0.05, ^**^p < 0.01, ^***^p < 0.001.

### Mediating roles of perceived social support and academic pressure

**Figure 2:**
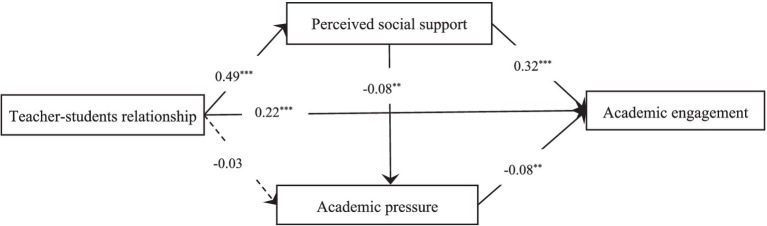
Results of M1 ^**^p < 0.01, ^**^p < 0.001.

SEM was performed to analyze the mediating roles of perceived social support and academic pressure in the association between teacher–student relationship and academic engagement (Figure 2). Gender, registered residence, age, and annual household income were coded as control variables and included in the analysis model (M1).

The results revealed that the analysis model achieved favorable fit indices (*χ^2^* = 26.53, *df* = 8, *p* < 0.001, RMSEA = 0.047, TLI = 0.93, and CFI = 0.97). Moreover, the model indicated that teacher–student relationship positively associated perceived social support [*β* = 0.49, standard error (SE) = 0.04, *p* < 0.001] and academic engagement (*β* = 0.22, SE = 0.06, *p* < 0.001). Perceived social support negatively associated academic engagement (*β* = 0.32, SE = 0.07, *p* < 0.001) and academic pressure (*β* = −0.08, SE = 0.01, *p* < 0.01). In addition, academic pressure negatively associated academic engagement (*β* = −0.08, SE = 0.03, *p* < 0.01). To examine the mediating roles of perceived social support and academic pressure in the association between teacher–student relationship and academic engagement, bootstrapping was conducted. According to the literature, if the 95% confidence level (CI) does not include 0, then the mediating role is significant at the 0.05 level (Shrout and Bolger, 2002). The results revealed that teacher–student relationship positively associated academic engagement through the mediating role of perceived social support (95% CI: 0.215–0.315) and through the combined mediating role of perceived social support and academic pressure (95% CI: 0.010–0.015). Therefore, H1, H2, and H4 were supported.

### Supplementary analyses

The present study also tested the model of academic stress might lead to poor teacher-student relationship and bias the perception of social support, which eventually associated academic engagement (M2). Although the M2 achieved favorable fit indices (*χ^2^* = 41.66, *df* = 8, *p* < 0.001, RMSEA = 0.064, TLI = 0.88, and CFI = 0.95), the data of present study tended to fit M1 better and therefore the present study just illustrated the results of M1.

## Discussion

This study found that teacher–student relationship positively associated university students’ academic engagement. Moreover, this study revealed that perceived social support and academic pressure mediated the association between teacher–student relationship and university students’ academic engagement. These findings could enhance our understanding of the mechanisms underlying the effect of teacher–student relationship on university students’ academic engagement, which filled the research gap. Additionally, these results further verified and expanded self-determination theory that positive teacher–student relationships was helpful to satisfy individuals’ basic needs of social support, with the satisfied basic needs of social support, university students could overcome the academic pressure and increase the level of academic engagement. Such results also pointed out that the interventions for improving university students’ academic engagement could aim at building positive teacher–student relationships.

### Effect of teacher–student relationship on academic engagement

This study revealed that teacher–student relationship positively associated university students’ academic engagement. Previous studies have explored the association between teacher–student relationship and academic engagement among primary or middle school students (Roorda et al., 2011; Chen et al., 2021; Cole-Lewis et al., 2023), and the present study extended this examination to university students. According to expected value theory, students’ learning is associated with their teachers’ expectations. When teachers hold positive expectations for specific students, these students tend to align their learning behaviors with such expectations (Saaty, 1986; Liu et al., 2014). Accordingly, if teachers expect their students to invest considerable time in their studies, these students are likely to demonstrate increased levels of academic engagement. Numerous studies have suggested that a positive teacher–student relationship can foster teachers’ positive expectations (e.g., Martin and Dowson, 2009; Xiong et al., 2020). This finding implies that a positive teacher–student relationship can enhance students’ academic engagement through the positive expectations of their teachers. Moreover, a positive teacher–student relationship offers students more opportunities to communicate with their teachers, thereby allowing the students to receive more guidance and encouragement.

### Mediating role of perceived social support

This study revealed that a positive teacher–student relationship positively associates university students’ academic engagement through the mediating role of perceived social support. Studies have reported that teacher–student relationship is positively associated with parent–child relationship and student–student relationship, which indicates that university students who have a positive relationship with their teachers perceive increased levels of social support from their teachers, parents, and classmates (Elffers et al., 2012; Elsaesser et al., 2018). Moreover, previous research has reported that higher levels of social support are associated with higher levels of academic engagement (Lam et al., 2012). According to self-determination theory (Ryan and Deci, 2000) and self-processing theory (Connell and Wellborn, 1991), greater perceived social support is beneficial for fulfilling students’ needs in terms of ability development and interpersonal relationships; such fulfillment can strengthen their learning motivation and trust in the learning process, ultimately enhancing their academic engagement. However, some studies have not identified association between perceived social support and academic engagement, and this discrepancy might be due to several factors (Shi, 2005; Cirik, 2015). First, different studies utilized different samples. For example, Shi (2005) included students with learning disabilities in their survey, whereas the present study included university students; such individual differences might have led to the inconsistent results. Second, the analysis methods varied between studies. For example, the present study used SEM, whereas Shi used linear regression analysis; this variation in analysis methods might also account for the differing outcomes. Therefore, additional studies are warranted to verify the findings of the present study.

### Combined mediating role of perceived social support and academic pressure

The results of this study reveal that teacher–student relationship can enhance students’ academic engagement through the combined mediating role of perceived social support and academic pressure. Conservation of resources theory explains the mechanism of pressure generation (Hobfoll, 2011). This theory states that all conditions aiding individuals in achieving their goals can be considered resources. When individuals perceive resource loss or a lack of expected returns from their investments, they experience stress (Liao et al., 2022). According to the aforementioned theory, strong social support represents a wealth of interpersonal resources, and when individuals perceive these resources as being excessively expended in pursuit of academic goals, they are likely to experience heightened academic pressure. By contrast, when individuals perceive an increase in social support resources, they are likely to experience a decrease in academic pressure. Studies have demonstrated that reducing academic pressure can enhance university students’ intrinsic learning motivation and commitment, thereby enhancing their academic engagement (Callicott and Park, 2003; Linnenbrink-Garcia and Pekrun, 2011; Zhang, 2014). Therefore, perceived social support can increase academic engagement by mitigating academic pressure. In educational settings, teachers often serve as crucial sources of social support for students, and a harmonious teacher–student relationship can further enhance this support (Elffers et al., 2012; Elsaesser et al., 2018). Accordingly, perceived social support and academic pressure are crucial mediators in the effect of teacher–student relationship on academic engagement.

## Limitations and future research directions

Although the present study tested the association between teacher–student relationship and university students’ academic engagement and assessed the mediating roles of perceived social support and academic pressure in this relationship, it has several limitations that warrant attention in future research. First, this study used a cross-sectional design, which limited our ability to deduce causal inferences. Therefore, future studies should consider using experimental or longitudinal designs to verify the findings of the present study. Second, the research sample of this study comprised only Chinese university students. Therefore, we could not test cultural differences across different countries. Numerous studies have revealed cultural differences in students’ academic engagement (e.g., Yamazaki, 2005; Joy and Kolb, 2009; Paloș et al., 2023), which might associate the associations between the variables examined in the present study. Therefore, future studies should collect data from diverse countries to ascertain any cultural differences in the associations between teacher–student relationship, perceived social support, academic pressure, and academic engagement. Finally, this study used self-report questionnaires, which might have led to common method bias. Although tests for common method variance did not indicate the existence of significant bias in this study, future studies can use multisubject reporting methods (such as parent-report, teacher-report, and peer-report questionnaires) for further mitigating the association of common method bias.

## Conclusion

On the basis of the findings of this study, we can draw the following conclusions. First, a positive teacher–student relationship can increase university students’ academic engagement. Second, a positive teacher–student relationship can increase university students’ academic engagement by increasing the level of perceived social support. Finally, a positive teacher–student relationship can increase university students’ academic engagement by increasing the level of perceived social support, which can reduce the level of academic pressure.

## Data availability statement

The raw data supporting the conclusions of this article will be made available by the authors, without undue reservation.

## Ethics statement

The studies involving humans were approved by This study was approved by the Human Research Ethics Committee of Shandong Normal University and conducted in accordance with the ethical principles of the Declaration of Helsinki and its later amendments. The studies were conducted in accordance with the local legislation and institutional requirements. The participants provided their written informed consent to participate in this study.

## Author contributions

XL: Conceptualization, Investigation, Methodology, Validation, Writing – original draft.
